# Fine‐scale hierarchical genetic structure and kinship analysis of the ascidian *Pyura chilensis* in the southeastern Pacific

**DOI:** 10.1002/ece3.5526

**Published:** 2019-08-05

**Authors:** Sarai Morales‐González, Emily C. Giles, Suany Quesada‐Calderón, Pablo Saenz‐Agudelo

**Affiliations:** ^1^ Instituto de Ciencias Ambientales y Evolutivas Facultad de Ciencias Universidad Austral de Chile Valdivia Chile; ^2^ Magister en Ciencias Mención Genética Escuela de Graduados Facultad de Ciencias Universidad Austral de Chile Valdivia Chile; ^3^ Doctorado en Ciencias Mención Ecología y Evolución Escuela de Graduados Facultad de Ciencias Universidad Austral de Chile Valdivia Chile

**Keywords:** Chile, fine spatial‐scale, hierarchical sampling, kinship, larval dispersal, relatedness, spatial autocorrelation

## Abstract

Studying population structure and genetic diversity at fine spatial scales is key for a better understanding of demographic processes that influence population connectivity. This is particularly important in marine benthic organisms that rely on larval dispersal to maintain connectivity among populations. Here, we report the results of a genetic survey of the ascidian *Pyura chilensis* from three localities along the southeastern Pacific. This study follows up on a previous report that described a genetic break in this region among localities only 20 km apart. By implementing a hierarchical sampling design at four spatial levels and using ten polymorphic microsatellite markers, we test whether differences in fine‐scale population structure explain the previously reported genetic break. We compared genetic spatial autocorrelations, as well as kinship and relatedness distributions within and among localities adjacent to the genetic break. We found no evidence of significant autocorrelation at the scale up to 50 m despite the low dispersal potential of *P. chilensis* that has been reported in the literature. We also found that the proportion of related individuals in close proximity (<1 km) was higher than the proportion of related individuals further apart. These results were consistent in the three localities. Our results suggest that the spatial distribution of related individuals can be nonrandom at small spatial scales and suggests that dispersal might be occasionally limited in this species or that larval cohorts can disperse in the plankton as clustered groups. Overall, this study sheds light on new aspects of the life of this ascidian as well as confirms the presence of a genetic break at 39°S latitude. Also, our data indicate there is not enough evidence to confirm that this genetic break can be explained by differences in fine‐scale genetic patterns among localities.

## 1 INTRODUCTION

Understanding the scale of population connectivity is perhaps one of the fundamental goals in the field of marine ecology. Overall, estimates of population connectivity can inform the design and management of conservation areas for protecting endangered or economically important species (Holland, Jenkins, & Stevens, 2017; Lowe & Allendorf, 2010; Selkoe & Toonen, 2011). In particular, studies that take into account fine geographic scales have been crucial for determining the genetic structure of natural marine populations and for inferring life history traits, such as dispersal and mating strategies (Costantini, Fauvelot, & Abbiati, 2007; Dahl, Pereyra, Lundälv, & André, 2012; Dupont, Viard, Dowell, Wood, & Bishop, 2009; Kovach, Breton, Berlinsky, Maceda, & Wirgin, 2010; Ledoux et al., 2010, 2012; Ni, Li, & Kong, 2011). Also, the inferences that can be obtained from fine‐scale spatial genetic structure analyses provide insights to propose management and conservation plans. In general, genetic surveys that consider small scales can shed light on the complex life histories of many marine species (Smouse, Peakall, & Gonzales, 2008), can help determine factors influencing genetic structuring, and can be used to define strategies for sustainable management and conservation (Costantini et al., 2007).

Life history characteristics influence the genetic structuring of marine invertebrate populations (Barbosa, Klanten, Puritz, Toonen, & Byrne, 2013; Bohonak, 1999; Hart & Marko, 2010; Pelc, Warner, & Gaines, 2009). Many marine invertebrates have a biphasic life cycle composed of a pelagic larval phase and a sessile adult stage (Pusack, Christie, Johnson, Stallings, & Hixon, 2014). Variation in larval dispersal and reproductive traits can affect the degree and scale of population genetic structure (Barbosa et al., 2013; Kamel, Hughes, Grosberg, & Stachowicz, 2012). For instance, various studies have shown that the absence (direct development) or the presence of a larval phase (indirect development) can be a strong determinant of the magnitude of genetic structure of populations of marine invertebrates (Goldson, Hughes, & Gliddon, 2001; Hoskin, 1997; Kamel, Grosberg, & Addison, 2014; Teske et al., 2007). Larval dispersal is a fundamental aspect of the life history of organisms with sessile adult stages. For instance, dispersal of propagules or larvae can facilitate escape from unfavorable local conditions (Kinlan & Gaines, 2003; Randolph & Steele, 1985; Vekemans & Hardy, 2004). Larval phases can last from minutes to months and depend on the diverse aspects of the life cycle of each species such as the duration of larval phases, mode of development, capacity for mobility, and sensorial abilities. Moreover, larval dispersal capacity can be affected by factors such as physical barriers, lack of habitats for settlement, among others (Cowen & Sponaugle, 2009; Selkoe, Henzler, & Gaines, 2008; Weersing & Toonen, 2009). That is, life history characteristics such as reproductive mode, and variables that affect dispersal are key in determining the spatial scale of genetic structure in marine invertebrates (Pusack et al., 2014).

Although dispersal delimits the connectivity of populations, other biological or environmental factors also play fundamental roles in shaping population structure (Barbosa et al., 2013; Guiñez et al., 2016; Kovach et al., 2010; Pelc et al., 2009; Vekemans & Hardy, 2004). For instance, the small‐scale geographic population structure of *Seriatopora hystrix* was studied by Maier, Tollrian, & Nürnberger, 2009. Despite the limited larval dispersal reported for this hermaphroditic coral, genetic differentiation between two sites in Egypt separated by 8 km was low. This observation could be due to abiotic forces or variation in the life history (selfing rates) between populations of this species. In another work, the genetic structure of *Mactra chinensis* was studied at fine spatial scales (Ni et al., 2011). The authors expected to find weak genetic structure at small scales since this bivalve is iteroparous, highly fertile and exhibits a planktonic larval phase of over 10 days. Interestingly, they found that genetic differentiation was high revealing the presence of cryptic barriers to gene flow. Taken together, these studies highlight the importance of conducting small‐scale genetic surveys, as these can improve our current understanding of the spatial scale at which populations are structured and reveal hidden factors that shape it.

Our study focuses on *Pyura chilensis*, an ascidian of the southeastern Pacific. In general, ascidians are hermaphroditic marine organisms that as adults are efficient sedentary filterers of small particles suspended in the water column (Lambert, 2007). Dispersal capacity of ascidians is influenced by reproductive mode and duration and mobility of larvae (Dupont et al., 2009). Some colonial ascidians have internal fertilization and brood their larvae, and settlement usually occurs near the mother colony. In contrast, solitary ascidians release their gametes into the water column and have higher dispersal potential as both eggs and larvae can be dispersed by currents (Petersen & Svane, 1995). *Pyura chilensis* Molina 1782 is a solitary ascidia found from low intertidal to subtidal zones (up to 70 meters deep) along the coast of Chile and Perú, between 10°S and 44°S (Lancellotti & Vasquez, 2000; Vásquez, 1983). This ascidian is an important economic resource for artisanal fishing, and in addition, it is ecologically important as individuals can form massive aggregates (Manríquez & Castilla, 2007) that provide habitat for a diverse community of many species (Manríquez & Castilla, 2007; Sepúlveda, Cancino, & Thiel, 2003). *P. chilensis* is a digonic hermaphrodite with external fertilization. Previous studies have suggested that larval duration in laboratory conditions is only 12 to 24 hr. After this time, the larva seeks a substrate on which to settle and metamorphose to its sessile adult life form (Cea, 1973). It has been documented that this species can self‐fertilize, but in the presence of congeners, cross‐fertilization is favored (Astorga & Ortiz, 2006; Manríquez & Castilla, 2005). In the last 20 years, populations of this species have been dramatically exploited (Davis, 1995). Previous studies have investigated the population genetic structure of *P. chilensis* using multiple markers such as allozymes (Astorga & Ortiz, 2006), nuclear and mitochondrial genes (Haye & Muñoz‐Herrera, 2013), and single nucleotide polymorphisms (Segovia, Gallardo‐Escárate, Poulin, & Haye, 2017). All of these previous studies have utilized samples taken hundreds of kilometers apart, but a recent study has shown that genetic differences exist between localities that are separated by only 20 km (Giles, Petersen‐Zúñiga, Morales‐González, Quesada‐Calderon, & Saenz‐Agudelo, 2017).

In this study, we investigate whether the observed population genetic structure of *P. chilensis* reported by Giles et al. (2017) in southern Chile may be due to life history traits such as dispersal and mating strategies. To do this, we conducted a genetic survey using a hierarchical experimental design and ten polymorphic microsatellite markers to assess genetic diversity. Our results confirm the presence of genetic differentiation between localities only 40 km apart. We found that in general individuals are randomly distributed at the scale up to 50 m. However, kinship analyses indicated the presence of sporadic self‐fertilization and cohesive dispersal events. The implications of the results found here for the conservation of this resource are also discussed.

## 2 MATERIALS AND METHODS

### 2.1 Sample collection

**Figure 1:**
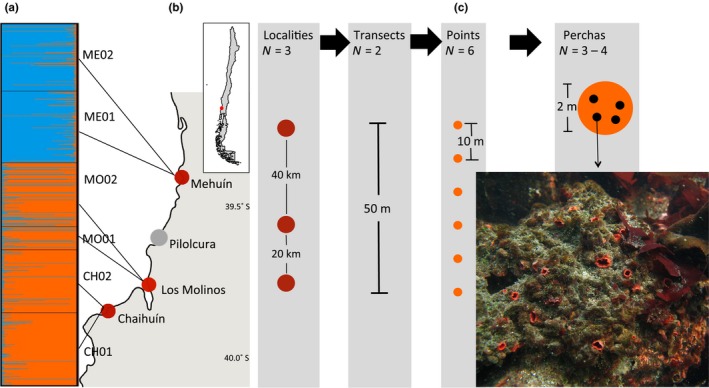
(a) Structure results for *K* = 2. (b) Map of the Coastal area in Chile indicating the sampling locations. The small insert on top indicates the location of the study area in Chile (red point). (c) Diagram of the sampling hierarchical sampling scheme used. Photograph of a group of *Pyura Chilensis* (percha) is shown on the bottom left. Photo credit: P. Saenz‐Agudelo

*Pyura chilensis* samples were collected from three localities along the southeast coast of Chile: Mehuín (ME), Los Molinos (MO), and Chaihuín (CH) (Figure 1) that span approximately 60 km of coastline. In each site, two transects of 50 m (ME01, ME02, MO01, M02, CH01, CH02) were established. In MO and CH, the transects were laid at a depth of 8–10 m on the rocky bottom; this was done by SCUBA diving. At Mehuín, transects were laid during low tide at the lower intertidal zone. Transects were laid using a measuring tape and weights. In each transect, six equidistant points were defined every 10 m (p00, p10, p20, p30, p40, p50). In each point, three to five “perchas” (natural aggregations of *P. chilensis*) were randomly collected within one meter of the measuring tape. Each percha can contain from a few to several dozens of individuals. We randomly sampled up to 16 individuals per percha that were preserved in 80% ethanol at 4°C. For each individual, we recorded its location along the corresponding transect. GPS coordinates from each transect were taken using a Garmin GPS.

### 2.2 DNA extraction and genotyping

DNA from a small portion of the tissue that forms the siphons was extracted using the HotSHOT protocol (Meeker, Hutchinson, Ho, & Trede, 2007). Ten polymorphic microsatellite regions developed previously for this species were amplified by PCR on a MultiGene OptiMax Thermal Cycler and run on an ABI 3500 fragment analyzer (Applied Biosystems) available at the AUSTRAL‐omics core facility of the Faculty of Science at the Universidad Austral de Chile (www.australomics.cl). For more information on the PCR protocol, marker details, and thermocycler profiles, see Giles et al. (2017). Samples were genotyped using Geneious v8.0.5 to determine the size of the alleles based on the GeneScan 500 LIZ standard (Applied Biosystems). Samples with more than two missing loci were removed from the data set.

### 2.3 Genetic diversity

Allele frequencies, sample size (*N*), number of alleles (Na), number of private alleles (PAP), observed (Ho), and unbiased expected heterozygosity (uHe) were estimated for each site and each locus using the software GenAlEx v6.503 (Peakall & Smouse, 2012). We used the same program to estimate the multilocus probability of identity (PI) and identity of siblings (PISibs) for the entire data set. Linkage disequilibrium between loci (LD), deviations from Hardy–Weinberg equilibrium (HWE), and the inbreeding coefficient (*F*_IS_) were determined using Genepop (Rousset, 2008). Markov chain parameters were as follows: 1,000 dememorizations, 100 batches, and 1,000 iterations per batch. We adjusted the *p*‐values using false discovery rate (FDR; Benjamini & Hochberg, 1995). We also used the software FreeNA to calculate the frequency of null alleles at each locus and to estimate whether F‐statistics were affected by the presence of null alleles (Chapuis & Estoup, 2007).

### 2.4 Population genetic structure

We used the program STRUCTURE v2.3 (Pritchard, Stephens, & Donnelly, 2000), which implements a Bayesian model based on groupings of individuals to determine the most likely number of populations (*K*) within our data set. Each population is characterized by a set of allele frequencies that are used to assign individuals to populations based on their genotypes. We ran STRUCTURE varying *K* from *K* = 1 to *K* = 10. For each *K*, the model was run ten times with a burn‐in of 200,000 iterations and 500,000 subsequent iterations. The most likely *K* was selected visually by inspecting the relationship between the maximum likelihood of the model given the data (LnP(D)) (Pritchard et al., 2000) and the second derivative of the rate of change of LnP(D) as described by Evanno (Evanno, Regnaut, & Goudet, 2005). Both metrics were compiled using STRUCTURE Harvester (Earl & VonHoldt, 2012).

Population genetic differentiation was determined using an analysis of molecular variance (AMOVA) implemented in GenAlEx v6.503. Population pairwise *F*_ST_ (Wright, 1951) was determined in GenAlEx v6.503, and the significance levels were adjusted using FDR correction after applying 9,999 permutations. Also, population pairwise *F*_ST_ was determined in FreeNA (Chapuis & Estoup, 2007), which uses the *ENA* correction method to provide a more accurate estimation of *F*_ST_ when the presence of null alleles is suspected. 10,000 replicates were used for bootstrapping the 95% confidence intervals.

### 2.5 Fine‐scale genetic structure

#### 2.5.1 Spatial autocorrelation

Spatial autocorrelation was determined at the scale up to 50 m within each of the six transects sampled at the three localities with the purpose of evaluating whether genetic similarity between individuals correlates with geographic distance between them (Smouse & Peakall, 1999). Six distance classes were assigned with class sizes of 10 m corresponding to distances between points along a transect. Genetic distance and geographic distance matrices were generated in GenAlEx v6.503, and the autocorrelation coefficient (*r*) was estimated for each class distance. We performed 9,999 random permutations and 10,000 bootstraps to determine the 95% confidence intervals around the null hypothesis of no spatial autocorrelation (*r* = 0). Significant positive *r* values for a given geographic distance indicate that samples in that distance are more closely related to each other than the average of all the transect samples. On the contrary, significant negative *r* values indicate that samples in that distance class are less closely related to each other than the average of all the transect samples.

#### 2.5.2 Relatedness and kinship analysis

We evaluated spatial patterns of genetic relatedness and kinship in three different ways. First, we compared average pairwise genetic relatedness at three hierarchical levels within each locality: (a) within each percha by locality, (b) within each point by locality, and (c) within each transect by locality. We evaluated whether average pairwise relatedness between individuals within a hierarchical level was different than the average relatedness across the entire data set at that hierarchical level. In other words, if dispersal is limited at one of these hierarchical levels, then one would expect to find higher mean relatedness among individuals at that level, compared to mean relatedness at the hierarchical level above. Genetic relatedness between pairs of individuals was generated in GenAlEx v6.503 using the Lynch and Ritland (1999) estimator. We calculated average relatedness within each level and performed 9,999 random permutations to determine the 95% confidence intervals around the null hypothesis of no difference across the hierarchical level (average relatedness = 0). Average relatedness at the percha level was determined including perchas that contained at least 10 individuals.

Second, we evaluated whether the proportion of full‐siblings differed among the various hierarchical levels. To do this, we calculated the probability that each pair of individuals were full‐siblings and compared this probability to the probability that the same pair of individuals was unrelated based on the allele frequencies of each locality and using a likelihood ratio test implemented in KINGROUP v2 (Konovalov, Manning, & Henshaw, 2004). A pair of individuals was accepted as being full‐siblings if the *p*‐value associated with the likelihood ratio test was ≤0.01. Significance levels for the pairwise likelihood values were estimated using 10,000 simulations for the null hypothesis of unrelated pairs of individuals and for the primary hypothesis of full‐sibling pairs. We then compared the proportion of full‐siblings within and among each hierarchical sampling level. To test whether proportions were significantly different, we used a two‐proportions Z test implemented in R v3.4.0 (R Core Team, 2017).

Finally, we evaluated whether the distribution of pairwise kinship coefficients (Loiselle, Sork, Nason, & Graham, 1995) differed within versus among localities. We used the kinship coefficients rather than relatedness coefficients for these analyses as these can be estimated with respect to the allele frequencies of the full data set. This allowed us to generate a metric that was based on all individuals in the data set and not only those from a specific locality (Iacchei et al., 2013; Meirmans & Van Tienderen, 2004). Kinship coefficients were calculated between each pair of individuals using GENODIVE (Meirmans & Van Tienderen, 2004). We then grouped kinship coefficients into bins and plotted the relative proportion of pairwise kinship values within and among localities for each bin class using R.

## 3 RESULTS

### 3.1 Genetic diversity

A total of 917 samples were genotyped, but from these 903 samples were successfully genotyped at eight or more loci and thus conform the final data set. All ten microsatellite loci were polymorphic in all samples, and on average, 21 alleles were found for each locus (Table 1). The number of private alleles at each site ranged from 1 (CH01 and CH02) to 17 (ME02). The observed heterozygosity values at each locus varied from 0.37 (PIU17) to 0.82 (PIU90), and unbiased expected heterozygosity ranged from 0.65 (PIU06) to 0.94 (PIU17). Five of ten loci showed significant deviations from HWE in all sites after FDR correction. Overall, heterozygote deficiency was detected in all sites. Estimates of *F*_IS_ were significantly different from zero in all sites, ranging from 0.18 (CH02) to 0.35 (ME02). We could not rule out that observed deviations from HWE were due to the presence of null alleles. Therefore, we conducted the genetic structure analyses with and without correction for null alleles. Significant genotypic linkage disequilibrium was found in 11 of 270 comparisons (4%), and only three comparisons remained significant after FDR correction, but no association was found across sites or loci. Interestingly, we found two pairs of individuals with identical multilocus genotypes found in Mehuín and Chaihuín and another four pairs of individuals with genotypes differentiated by only one loci found in Chaihuín. Since the multilocus probability of identity (PI) and identity of siblings (PISibs) in our data set were low (5.9E‐16 and 1.5E‐05, respectively), it is very likely that these individuals are the product of self‐fertilization.

**Table 1:** Summary of genetic variation at 10 microsatellite loci for six sampling sites of *Pyura chilensis*

Sites	ME01	ME02	MO01	MO02	CH01	CH02	Mean
Lat	−39.421007	−39.420479	−39.855822	−39.855507	−39.956580	−39.939650	
Lon	−73.218053	−73.218713	−73.393684	−73.394014	−73.598200	−73.591060	
*n*	178	164	59	160	182	160	
Locus							
PIU76							
*N*	112	107	53	143	169	144	121
Na	29	34	22	29	24	28	28
Ho	.22	.28	.51	.55	.69	.64	.48
uHe	.93	.94	.90	.92	.90	.91	.92
*F*_IS_	**.76**	**.70**	**.43**	**.41**	**.24**	**.30**	**.47**
PIU66							
*N*	176	161	59	159	178	157	148
Na	20	19	15	18	16	18	18
Ho	.63	.69	.51	.58	.51	.54	.57
uHe	.76	.79	.81	.79	.73	.73	.77
*F*_IS_	**.18**	.12	**.38**	**.26**	**.30**	**.26**	**.25**
PIU20							
*N*	173	164	59	160	180	160	149
Na	25	27	19	22	23	23	23
Ho	.82	.76	.76	.76	.81	.81	.78
uHe	.93	.93	.91	.92	.91	.91	.92
*F*_IS_	**.12**	**.19**	**.17**	**.18**	.11	**.12**	**.15**
PIU67							
*N*	176	163	59	157	179	157	149
Na	22	24	14	15	16	15	18
Ho	.79	.83	.78	.80	.82	.73	.79
uHe	.90	.88	.84	.87	.83	.82	.86
*F*_IS_	**.12**	.06	.07	.07	**.02**	**.11**	**.07**
PIU19							
*N*	174	162	58	158	181	155	148
Na	28	29	16	24	17	16	22
Ho	.82	.81	.78	.69	.76	.75	.77
uHe	.94	.95	.92	.92	.91	.90	.92
*F*_IS_	.14	**.15**	.15	**.25**	**.17**	**.17**	**.17**
PIU90							
*N*	176	164	59	160	182	160	150
Na	19	19	9	17	14	14	15
Ho	.84	.77	.81	.84	.79	.85	.82
uHe	.90	.89	.85	.86	.85	.85	.87
*F*_IS_	.07	**.14**	.05	.02	.07	.00	**.06**
PIU36							
*N*	127	142	50	135	111	127	115
Na	24	32	19	23	22	15	23
Ho	.46	.51	.58	.55	.62	.61	.55
uHe	.92	.92	.90	.88	.88	.89	.90
*F*_IS_	**.50**	**.45**	**.36**	**.38**	**.30**	**.32**	**.38**
PIU82							
*N*	173	160	54	150	161	144	140
Na	23	24	20	34	30	24	26
Ho	.50	.42	.33	.47	.35	.40	.41
uHe	.86	.84	.88	.90	.89	.88	.87
*F*_IS_	**.42**	**.50**	**.62**	**.48**	**.60**	**.55**	**.53**
PIU17							
*N*	135	129	55	156	177	154	134
Na	31	34	25	30	25	28	29
Ho	.31	.25	.38	.40	.44	.42	.37
uHe	.95	.94	.95	.94	.94	.95	.94
*F*_IS_	**.67**	**.74**	**.60**	**.57**	**.53**	**.56**	**.61**
PIU06							
*N*	153	148	58	156	182	159	143
Na	14	18	4	13	11	11	12
Ho	.51	.47	.81	.77	.77	.87	.70
uHe	.84	.88	.52	.59	.53	.55	.65
*F*_IS_	**.40**	**.47**	**−.57**	**−.30**	**−.46**	**−.60**	**−.18**
Multilocus							
*N*	158	150	56	153	170	152	140
Na	24	26	16	23	20	19	21
Ho	.59	.58	.63	.64	.66	.66	.62
uHe	.89	.90	.85	.86	.84	.84	.86
*F*_IS_	**.34**	**.35**	**.23**	**.23**	**.19**	**.18**	**.25**
PAP	11	17	3	5	1	1	

Note

Sample size (*N*), number of alleles (Na), number of private alleles (PAP), observed (Ho), and unbiased expected heterozygosity (uHe) were estimated for each site and each locus. *F*_IS_ values in bold indicate significant deviations from HWE after standard FDR correction. GPS coordinates were taken using a Garmin GPS.

### 3.2 Population genetic structure

Despite that the natural logarithm of the maximum likelihood score consistently increased as a function of *K* (Appendix S1), both using Evanno's delta *K* method and a visual inspection of the barplots for different values of *K* indicated that *K* = 2 was the most likely number of populations (Figure 1, Appendix S2). One genetic cluster clearly grouped samples from Mehuín, and the second cluster grouped those from Los Molinos and Chahuín. We also detected several individuals with high levels of admixture. Approximately 5% of individuals in Mehuín presented a genotype more similar to the cluster of Los Molinos and Chaihuín. Additionally, 17% of individuals from Los Molinos had a genetic composition more similar to that of the Mehuín cluster, but only 2% of individuals from Chaihuín had this genetic composition.

The AMOVA indicated that 2% (*p* < .001) of the total genetic variation occurred among localities, 31% (*p* < .001) was attributed to variation among individuals, and 67% (*p* < .001) occurred within individuals (Table 2). Additionally, population pairwise *F*_ST_ comparisons estimated with permutations revealed significant levels of genetic subdivision between sites ranging from 0.000 to 0.048 (Table 3). Population pairwise *F*_ST_ with accurate estimation using FreeNA was significant for all comparisons between Mehuín sites and the other sites and ranged from 0.026 to 0.041.

**Table 2:** AMOVA of genetic differentiation of *Pyura chilensis* sampled from the localities of Mehuín, Los Molinos, and Chaihuín

Source of variation	*df*	SS	MS	Est. Var.	% of variance	*F*‐statistics	Value	*p*‐value
Among Pops	5	187.262	37.452	0.107	2	*F*_ST_	.024	<.001
Among Indiv	897	5,078.239	5.661	1.351	31	*F*_IS_	.313	<.001
Within Indiv	903	2,672.500	2.960	2.960	67	*F*_IT_	.330	<.001

Note

*p*‐values were calculated from 9,999 permutations.

**Table 3:** Pairwise differentiation of *Pyura chilensis* populations using ten microsatellite markers

	ME02	ME01	MO02	MO01	CH02	CH01
ME02		**.002**	**.030**	**.034**	**.040**	**.048**
ME01	.001		**.032**	**.037**	**.042**	**.046**
MO02	**.027**	**.026**		.001	.002	**.005**
MO01	**.034**	**.033**	.001		**.000**	**.004**
CH02	**.038**	**.038**	.002	.000		**.002**
CH01	**.041**	**.039**	.002	.001	.000	

Note

Above the diagonal are *F*_ST_ values obtained by permutations in GenAlEx. Below the diagonal are *F*_ST_ values calculated using the ENA method to correct the effect of the presence of null alleles. Values in bold indicated significant values (*α* = .05) after FDR correction.

### 3.3 Fine‐scale genetic structure

#### 3.3.1 Spatial autocorrelation

**Figure 2:**
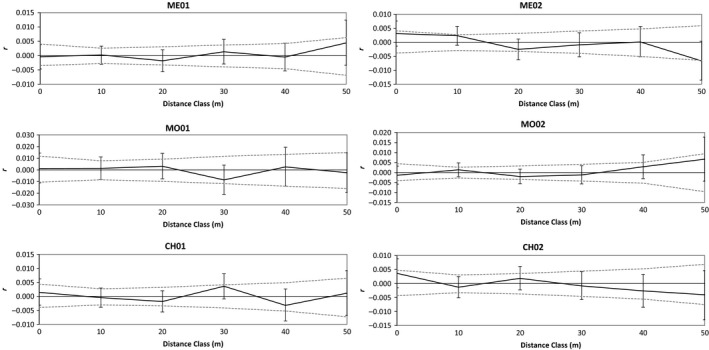
Small‐scale spatial autocorrelations. Correlation coefficients between genetic and geographic distance at the scale up to 50 m and distance class sizes of 10 m were calculated for each transect by locality. The dashed gray lines show 95% confidence intervals around the null hypothesis of no spatial structure (*r* = 0), and error bars indicate the 95% confidence intervals around the correlation for each distance class

The spatial autocorrelation analysis for each transect of each locality showed no significant autocorrelation at any distance at the scale up to 50 m (Figure 2). Overall, this analysis suggests that at the scale up to 50 m, we cannot reject the hypothesis of a random distribution of genotypes regardless of the locality.

#### 3.3.2 Relatedness and kinship analysis

**Figure 3:**
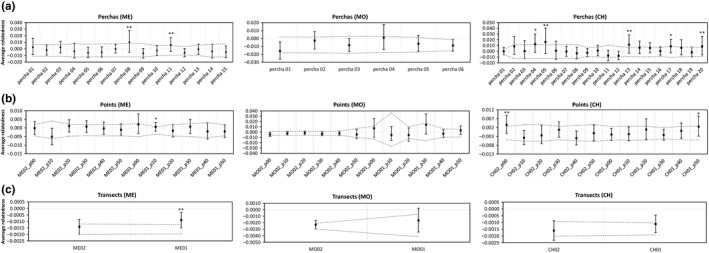
Average relatedness (Lynch and Ritland estimator) at three hierarchical levels: (a) within each percha by locality, (b) within each point by locality, and (c) within each transect by locality. The black quadrates represent mean relatedness within hierarchical level. The upper and lower error bars indicate 95% confidence intervals around the average relatedness calculated with within each sample by bootstrapping. The gray dashed lines indicate the 95% confidence limits of the null hypothesis of no difference across samples at a given hierarchical level. One asterisk indicates significant mean relatedness with *α* = .05 and, two asterisks, *α* = .01

We examined the average relatedness coefficients at different hierarchical levels. We found that mean pairwise relatedness within perchas was, in some cases, significantly higher than the mean relatedness of the transect to which perchas belonged (Figure 3). Significant within percha mean relatedness was found in 2 of 15 perchas analyzed in Mehuín (percha 08 of ME02 and percha 11 of ME01; *p* ≤ .01) and within 5 of 20 perchas in Chaihuín (percha 05 of CH02, percha 13 of CH01, and percha 20 of CH01 *p* ≤ .01; percha 04 of CH02 and percha 17 of CH01 *p* ≤ .05). For Los Molinos, the average relatedness coefficients of the six perchas analyzed were not significantly different from random expectations. We also evaluated the average relatedness coefficients at higher hierarchical levels. For instance, observed average relatedness coefficients were also higher than expected by chance in some cases when all samples were grouped by points [point 10 of ME01 (*p* ≤ .05), point 00 of CH02 (*p* ≤ .01), and point 50 of CH01 (*p* ≤ .05)]. At the higher level (transect), only one average relatedness coefficient was significantly different than expected by chance, ME01 (*p* ≤ .01). However, this coefficient was negative and close to zero, indicating that the average relatedness within ME01 was lower than mean relatedness across both Mehuín transects.

**Figure 4:**
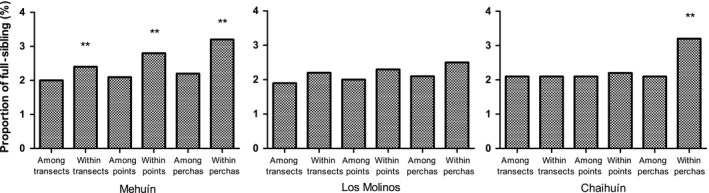
Proportion of full‐sibling pairs by locality within and among each sampling hierarchical level. Two asterisks indicate that the comparison between proportions of full‐siblings was significantly different (*p* < .01) in this hierarchical level

We analyzed the proportion of full‐siblings at the different hierarchical levels. The highest proportion of full‐sibling pairs was found within perchas for all localities, ranging from 2.5% to 3.2% (Figure 4). The proportion of full‐siblings within points ranged from 2.2% to 2.8% and within transects ranged from 2.1% to 2.4%. In Mehuín, the proportions of full‐siblings within perchas, points, and transects were significantly higher than among perchas, points, and transects (perchas *χ* ^2^ = 11.4, *df* = 1, *p* < .1; points *χ* ^2^ = 15.9, *df* = 1, *p* < .1; transects *χ* ^2^ = 17.7, *df* = 1, *p* < .1). For Los Molinos, the proportions of full‐siblings were not significantly different at any hierarchical level (perchas *χ* ^2^ = 1.4, *df* = 1, *p* = .1; points *χ* ^2^ = 2.0, *df* = 1, *p* = .08; transects *χ* ^2^ = 3.7, *df* = 1, *p* = .03). For Chaihuín, the proportion of full‐siblings within perchas was significantly higher than among perchas (*χ* ^2^ = 22.3, *df* = 1, *p* < .1). In the other two hierarchical levels, there are no significant differences (points *χ* ^2^ = 1, *df* = 1, *p* = .2; transects *χ* ^2^ = .7, *df* = 1, *p* = .2).

**Figure 5:**
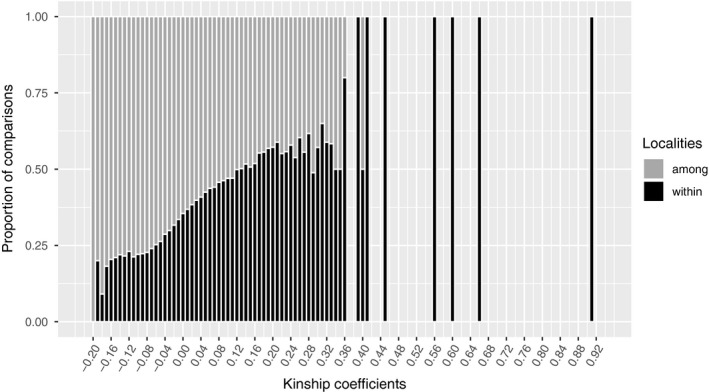
Proportion of pairwise kinship comparisons within localities and among localities. Kinship coefficients were divided into 0.01 bins (each vertical line), and each value is partitioned comparing the proportion within localities (dark bars) and among localities (gray bars). Absence of bars indicates kinship coefficients that were not found in our data set

Finally, we also examined how kinship coefficients were distributed within and among localities. Overall kinship coefficients ranged from −0.19 to 0.91 within localities and from −0.20 to 0.40 among localities (Figure 5). Interestingly, pairwise kinship coefficient values lower than 0.25 (full‐siblings; Loiselle et al., 1995) were found in greater proportions among localities than within localities. Kinship coefficients between 0.25 and 0.34 were nearly as frequent within as among localities. Finally, kinship coefficients higher than 0.36 were rare (10 pairs) and found almost exclusively within two localities (two pairs in Mehuín and six pairs in Chaihuín) and even within the same percha, suggesting that there are individuals that are nearly identical and that settle within a few centimeters of one another.

## 4 DISCUSSION

In the past several decades, genetic studies conducted at fine spatial scales have shown that many marine organisms are not distributed randomly at small scales despite high potential for larval dispersal (Kamel et al., 2012; Ni et al., 2011). Moreover, these studies have helped to infer patterns of life history traits of marine species that could not be obtained otherwise. For example, inferences of dispersal capacity and mating strategies have been obtained from fine‐scale population genetic studies of *Corallium rubrum* (Costantini et al., 2007; Ledoux et al., 2010), *Stylissa carteri* (Giles, Saenz‐Agudelo, Hussey, Ravasi, & Berumen, 2015), and *Cominella maculosa* (Dohner, Phillips, & Ritchie, 2018). Here, using a hierarchical sampling design and ten polymorphic microsatellite markers, we have uncovered some important aspects of the life history characteristics of a keystone tunicate found off the coast of Chile. First, despite its low dispersal potential according to inferences of its pelagic larval duration (PLD) in laboratory conditions, we found that on average, larval dispersal of *P. chilensis* does not appear to be restricted to small distances (<50 m). Second, we found that occasionally, related individuals settle together or in very close proximity. Third and last, we confirm the presence of a genetic break between −39.42°S and −39.85°S latitude. Our data indicate there is no evidence to suggest that this break can be explained by differences in fine‐scale population structure; thus, we open the avenue for further studies that can help understand the nature of this genetic discontinuity.

Given the life history characteristics of this species, that is, having a short pelagic larval duration (PLD) characteristic of low dispersal capacity (Cea, 1973), we expected to find a spatial distribution of genotypes on the scale up to 50 m as previous studies of other ascidians have found (David, Marshall, & Riginos, 2010; Dupont et al., 2009; Ordóñez, Pascual, Rius, & Turon, 2013). Contrary to our expectations, we could not reject the hypothesis that genotypes are randomly distributed up to this scale. For instance, a study of *Styela plicata* (David et al., 2010), a solitary hermaphroditic ascidian with a similar PLD to *P. chilensis* (1–2.5 days), has provided evidence of spatial genetic structure between individuals that were only 0–5 m apart. Interestingly, however this fine‐scale spatial genetic structure was only found for individuals at low latitudes, while individuals at higher latitudes were randomly distributed at the scale up to 60 m. In contrast, a study of the solitary ascidian *Microcosmus squamiger* found a lack of genetic differentiation among localities separated by tens of kilometers. Here, the authors hypothesize that this could be due to the naturally active dispersal capabilities of this species or due to the two large commercial ports, north and south of the studied coastline, that likely have contributed to dispersal of *Microcosmus squamiger* (Ordóñez et al., 2013). These contrasting results indicate that although dispersal capacity can be an important determinant of the connectivity of marine populations, other factors such as habitat continuity (Pinsky, Palumbi, Andréfouët, & Purkis, 2012) or dispersal by means of anthropogenic vectors (Teske, 2014) also play fundamental roles in the structuring of populations.

Here we have found that although *P. chilensis* has a wide geographic distribution (Lancellotti & Vasquez, 2000; Vásquez, 1983), this species can form groups of relatives that appear to settle together. We found evidence of several pairs of full‐siblings within the same perchas and as far as 10 m apart. Interestingly, we found that in the locality of Mehuín, the number of first‐degree relatives increased as the distance between pairs of individuals decreased. Also, we found that average pairwise genetic relatedness did not always follow random expectations at the scale of perchas and points for Mehuín and Chaihuín. However, results from the spatial autocorrelation analyses indicated no spatial correlation at this scale. Taken together, these results indicate that the hypothesis of "limited larval dispersal" explains the few cases of higher than expected kinship found at small scales (<50 m). Other phenomena such as larval cohesion and/or variability in reproductive success could explain these patterns of family structure as has been suggested previously (Broquet, Viard, & Yearsley, 2013; D'Aloia & Neubert, 2018).

Contrary to the belief that there is a high degree of mixing of genotypes in the ocean due to oceanographic circulation, long PLDs, and high larval mortality (D'Aloia & Neubert, 2018), there is also evidence that some marine organisms form kin aggregations like *Crassostrea virginica* (Adrian, Lack, & Kamel, 2017), *Panulirus interruptus* (Iacchei et al., 2013), and *Coryphopterus personatus* (Selwyn et al., 2016). Several explanations exist for how highly related individuals can be found at small spatial scales. First, larval retention or larval cohorts can disperse in the plankton as clustered groups (Ben‐Tzvi et al., 2012; Bernardi, Beldade, Holbrook, & Schmitt, 2012). Second, adult immobility and gametes with short lifetimes favor inbreeding and related individuals to be clustered at small spatial scales (Costantini et al., 2007; Ledoux et al., 2010). Third, genetic drift can alter the effective size of local breeding groups and variance in reproductive success among individuals (Broquet et al., 2013; D'Aloia & Neubert, 2018). While our data are insufficient to evaluate the influence of genetic drift over the formation of kin aggregations, we speculate that larval cohesion or occasional limited dispersal leads to the aggregation of relatives. This is because at the scale up to 50 m, aggregations of relatives were found within perchas and points, but limited dispersal was not found up to this scale. That is, larvae likely disperse at distances >50 m, but they do so cohesively. Therefore, they settle together or close to one another. Perhaps additional studies involving spatiotemporal sampling with larger continuous transects could help to elucidate the drivers of marine kin structure. Finally, recent reviews have shown that there is high incertitude in assigning relationships among individuals when using small microsatellite panels (D'Aloia, Xuereb, Fortin, Bogdanowicz, & Buston, 2018). Therefore, it is important to note that our results and their interpretation are bounded to this limitation and should be interpreted with caution.

Furthermore, our results reveal another important aspect of the biology of *P. chilensis*. We did find two pairs of individuals with identical multilocus genotypes and another four pairs of individuals with genotypes differentiated by only one locus. In addition, we found systematic deviations of HWE in all sites. While we cannot completely rule out the potential influence of null alleles, these results provide the first indirect evidence that self‐fertilization and mating between related individuals might be common in natural populations of this species. This has also been observed in laboratory conditions where groups of sexually mature individuals were assigned to reproductive isolation for different periods. Manríquez and Castilla (2005) have shown that self‐fertilization is more frequent when individuals are exposed to extended period of reproductive isolation.

Previous studies that have characterized the population structure of this species have done so at much larger spatial scales. In a study where enzymatic markers were used, weak population structuring of *P. chilensis* is reported between Puerto Montt (41°S) and two sites to the north, Talcahuano (36°S) and Antofagasta (23°S) (Astorga & Ortiz, 2006). Later, Haye and Muñoz‐Herrera (2013), using a nuclear and a mitochondrial marker, describe genetic homogeneity for this organism along practically the entire southeast Pacific (from 26°S to 41°S) with the exception of one locality where individuals are genetically distinct (Los Molinos, included in this study). Three well‐differentiated mitochondrial clades have also been found for *P. chilensis*, and one of these is only found in Los Molinos. More recently, three genetically distinct groups along the southeastern Pacific have been identified according to single nucleotide polymorphisms and Los Molinos has been highlighted again as the location where the highest degree of genetic differentiation occurs (Segovia et al., 2017). Our results and those from Giles et al. (2017) indicate that Los Molinos is not an outlier locality but rather a site in close proximity to a pronounced genetic break. Our results show that the break is located at a narrow section of the coastline between Mehuín and Pilolcura (both 39°S). These two locations are separated by a distance of only 20 km. Further studies are required, though, to evaluate the nature of this genetic break, the geographic extension of the two genetic clades we have reported, and how all of this fits in the context of previous large‐scale studies.

Moreover, our results indicate that individuals from the locality of Mehuín are genetically different from individuals from two other locations south of Mehuín. While Mehuín was the only site where samples were collected from the intertidal zone, it should be noted that no genetic differences have been found between intertidal and subtidal samples from Mehuín or other localities where *P. chilensis* is still found at the intertidal zone (unpublished results). As such, it is unlikely that differences in sampling zones between localities explain the genetic differentiation found. Additionally, the genetic differentiation of Mehuín does not appear to be a product of limited larval dispersal at least at the scale of 40 km because genetic homogeneity was found between the localities of Pilolcura and Chaihuín (Giles et al., 2017) as well as between Mehuín and other localities north of it (unpublished data). Rather, our results indicate the presence of a barrier to gene flow between Mehuín and Pilolcura. Genetic breaks close to 39°S latitude have been reported for other species such as the red alga *Mozzarella laminariales* (Montecinos et al., 2012), the barnacle *Notochthamalus scabrosus* (Ewers‐Saucedo et al., 2016), and the bivalve *Perumytilus purpuratus* (Guiñez et al., 2016). There is evidence of a physical oceanographic discontinuity of coastal waters from 36°S to 40°S (Atkinson et al., 2002). This area is characterized by presenting variation in sea surface temperature, in stratification, and flow of currents. We can only speculate that this reported environmental heterogeneity could be responsible for the observed genetic break. Further studies that include higher genetic resolution such as genome scans and detailed sampling schemes covering larger proportions of the Chilean coastline are required to determine what factors are causing the genetic break near Mehuín.

In summary, we confirm the presence of a genetic break of *P. chilensis* near 39.5°S. Our results indicate that this genetic discontinuity cannot be explained by differences in local population processes and seems to be rather the consequence of local circulation features and/or environmental heterogeneity. We have also found evidence of aggregations of relatives at small spatial scales, which indicates that the spatial distribution of related individuals can be nonrandom at small spatial scales (<1 km) and which suggests that dispersal might be occasionally limited in this species (D'Aloia & Neubert, 2018).

Since the larval dispersal potential of *P. chilensis* appears to be greater than that estimated in laboratory conditions, sampling that considers larger transects such as 100–200 m could help to better characterize dispersal of this organism. In this way, more optimal conservation management could be designed if significant intercepts are obtained from spatial autocorrelation analyses (Brauer, Unmack, Hammer, Adams, & Beheregaray, 2013; Campos Telles, Guedes Coelho, Chaves, Diniz‐Filho, & D'Ayala Valva, 2003; Chung et al., 2006; Diniz‐Filho & Campos Telles, 2002; Hrbek, Crossa, & Farias, 2007). Finally, with the genetic information obtained from this study and considering that this organism is an important economic and ecological resource, we recommend that conservation programs target the two genetic populations that are located north and south of the locality of Mehuín.

## CONFLICT OF INTEREST

The authors declare no conflict of interest.

## AUTHOR CONTRIBUTIONS

PSA conceived the study; PSA, SMG, EG, and SQC conducted sampling; SMG performed molecular analyses; SMG and PSA analyzed and interpreted the data; SMG wrote the first draft of the manuscript and all co‐authors contributed. This study is part of the Master's thesis of SMG.

## Data Availability

Individual multilocus microsatellite genotypes are deposited in Dryad: https://doi.org/10.5061/dryad.g5b5q70
