# Supplementary material for: Fine‐scale hierarchical genetic structure and kinship analysis of the ascidian Pyura chilensis in the southeastern Pacific
Source: Ecol Evol. 2019 Aug 5;9(17):9855–68. doi: 10.1002/ece3.5526 (PMC6745665; doi:10.1002/ece3.5526)
Supplement: Supplementary file 1 [file ECE3-9-9855-s001.docx]

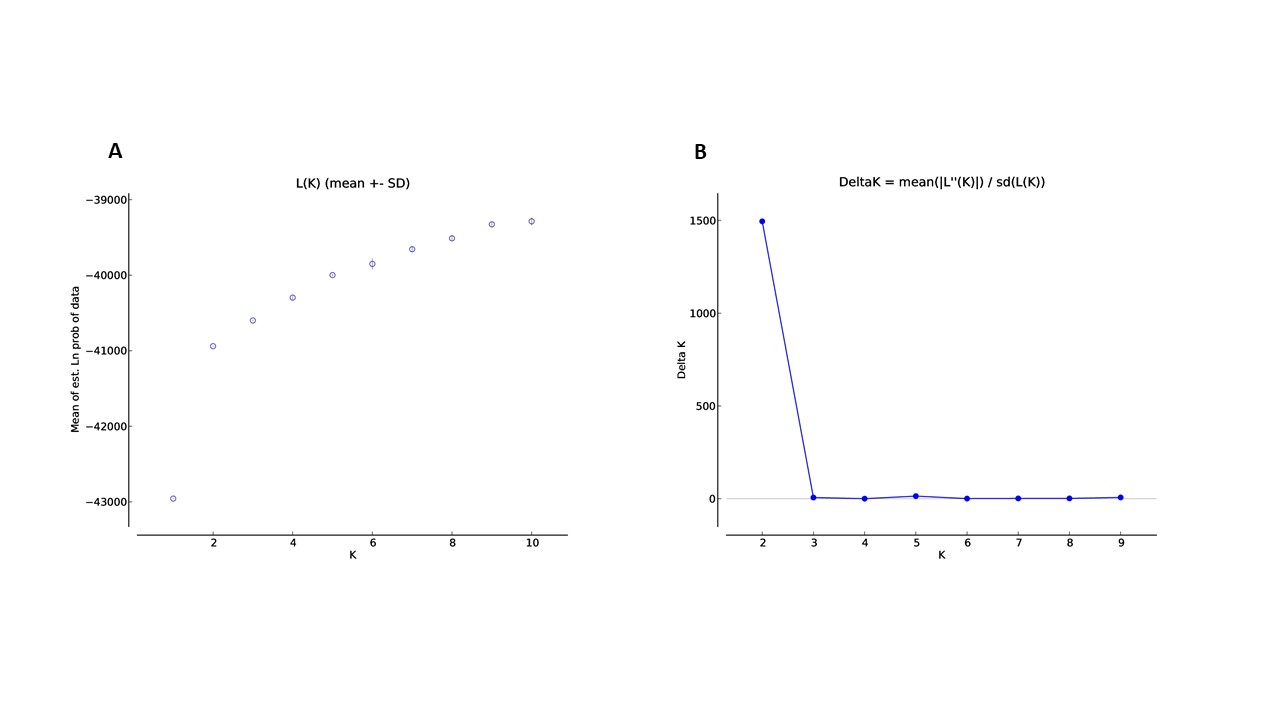
**Appendix S1.** A) Plot of mean likelihood L(K) per K value from STRUCTURE. B) Plot of Delta K according to Evanno (2005) as a function of K.


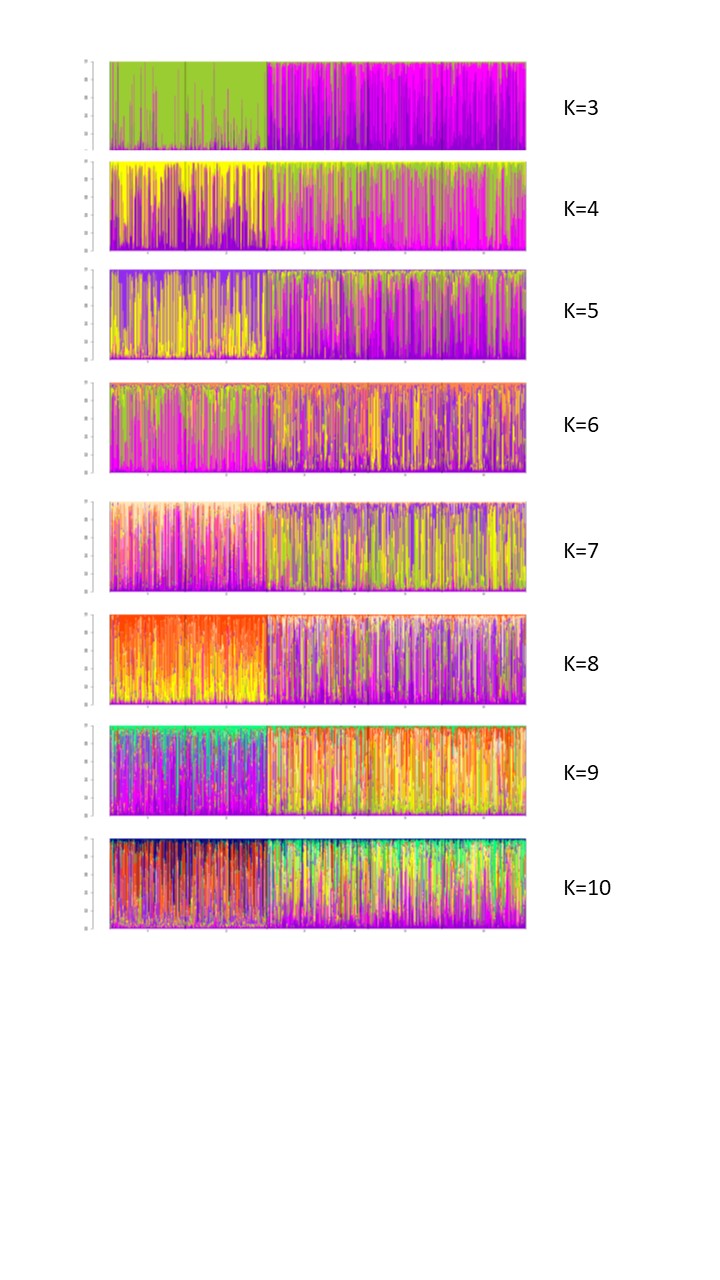


**Appendix S2.** Structure results for K=3 to K=10.
